# Spontaneous complete regression of pancreaticoduodenal artery aneurysms with celiac artery occlusion after aorto-splenic bypass without additional treatment: a case report

**DOI:** 10.1186/s40792-024-01880-3

**Published:** 2024-04-08

**Authors:** Sho Fujiwara, Keiichiro Kawamura, Yoshiyuki Nakano, Tetsuo Watanabe, Hiroshi Yamashita

**Affiliations:** 1 Department of Surgery, Iwate Prefectural Chubu Hospital, 17-10 Murasakino, Kitakami, Iwate 024-8507 Japan; 2 Department of Surgery, Columbia University Irving Medical Center, 622 West 168th St, New York, NY 10032 USA; 3 Department of Vascular Surgery, Iwate Prefectural Isawa Hospital, 61 Ryugababa, Mizusawa, Oshu, Iwate 023-0864 Japan; 4 Department of Vascular Surgery, Japan Community Health Care Organization Sendai Hospital, 2-1-1 Murasakiyama, Sendai, Miyagi 981-3205 Japan; 5 Department of Cardiovascular Surgery, Sendai City Hospital, 1-1-1 Asutonagamachi, Sendai, Miyagi 982-8502 Japan; 6 Department of Vascular Surgery, Kitakami Saiseikai Hospital, 15-33 Kunenbashi, Kitakami, Iwate 024-0063 Japan

**Keywords:** Pancreaticoduodenal artery aneurysm, Pancreaticoduodenal artery arcade, Bypass surgery, Endovascular embolization

## Abstract

**Background:**

Pancreaticoduodenal artery aneurysm (PDAA) is a rare, but fatal disease. However, the association between aneurysm size and the risk of rupture remains unclear. There are many options for therapeutic strategies that should be discussed well because the treatment options are often complicated and highly invasive. However, it remains unclear whether additional endovascular therapy is essential for all patients undergoing bypass surgery. Here, we present a case of triple PDAAs with celiac axis occlusion and spontaneous complete regression of inferior PDAAs (IPDAA) after aneurysmectomy of superior PDAA (SPDAA) and aorto-splenic bypass.

**Case presentation:**

A 68-year-old woman presented with one SPDAA and two IPDAAs caused by celiac axis occlusion. Aneurysmectomy for IPDAAs was difficult because of their anatomical location and shape. Therefore, we planned a two-stage hybrid therapy. The patient underwent aorto-splenic bypass and resection of the SPDAA. Follow-up CT was performed to evaluate the IPDAAs before planned endovascular embolization. Spontaneous regression of the IPDAAs and normalized PDA arcade decreased the blood flow in the PDA arcade. The patient is doing well without graft occlusion, and the IPDAAs have completely regressed 7 years after surgery.

**Conclusion:**

Normalization of hyperinflow to the PDA arcade can lead to the regression of PDAA. Potentially, additional endovascular therapy may not be required in all cases when dilation of the PDA improves. However, more cases must be accumulated to establish criteria for predicting the risks of short- and long-term PDAA ruptures.

## Introduction

Pancreaticoduodenal artery aneurysms (PDAA) are rare, accounting for only 2% of all splanchnic artery aneurysms [1]. Diagnosis is difficult because PDAA is often asymptomatic or presents with unspecific symptoms [1, 2]. Although rupture of PDAAs has a high mortality rate, the association between aneurysm size and the risk of rupture has not been clarified, unlike that in other aortic aneurysms. Many PDAAs are caused by occlusion or stenosis of the celiac artery, increasing the pancreaticoduodenal artery (PDA) arcade blood flow [3–5]. Therapeutic strategies include surgery, endovascular embolization, and hybrid therapy [6–9]. There are many options for therapeutic strategies, and they should be discussed well because the treatment options are often complicated depending on the medical facility and condition of the aneurysm.

Here, we present a case of a superior PDAA (SPDAA) and two inferior PDAAs (IPDAA) with celiac axis occlusion and spontaneous complete regression of the IPDAAs after aneurysmectomy of the SPDAA and aorto-splenic bypass.

## Case presentation

**Fig. 1:**
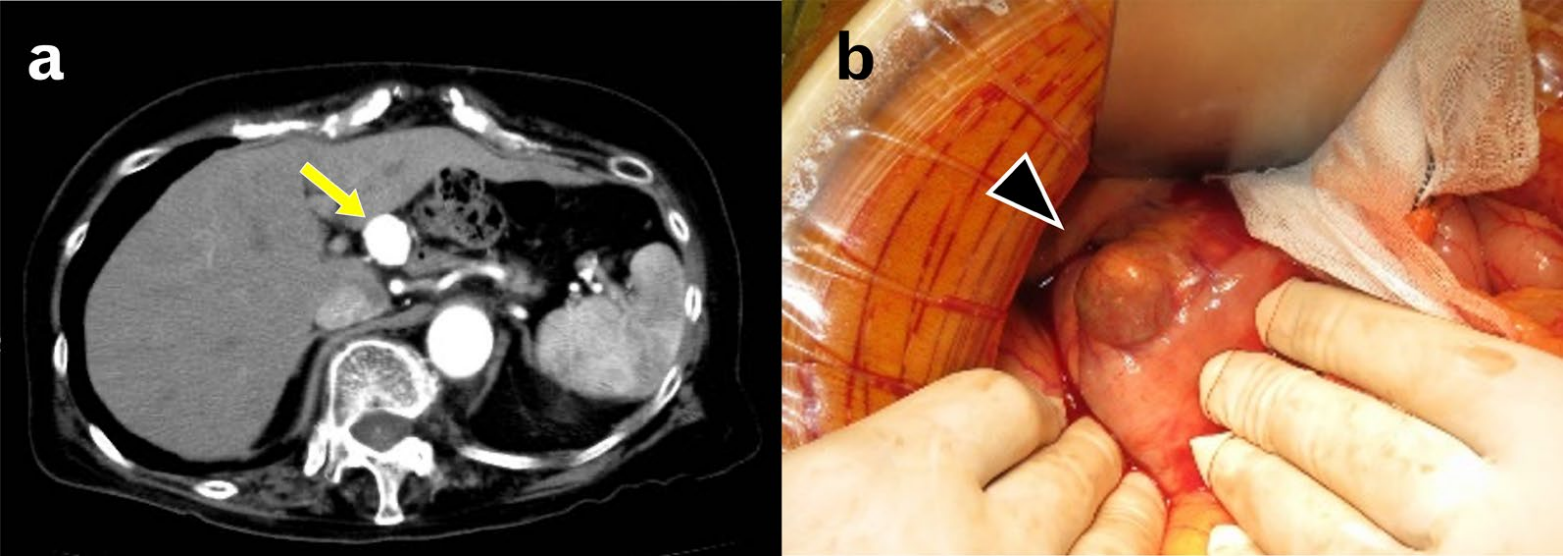
Preoperative contrast-enhanced computed tomography and intraoperative image.** a** 20 × 17 mm superior pancreaticoduodenal artery aneurysm (yellow arrow). **b** Intraoperative finding of a superior pancreaticoduodenal artery aneurysm (black arrowhead)

**Fig. 2:**
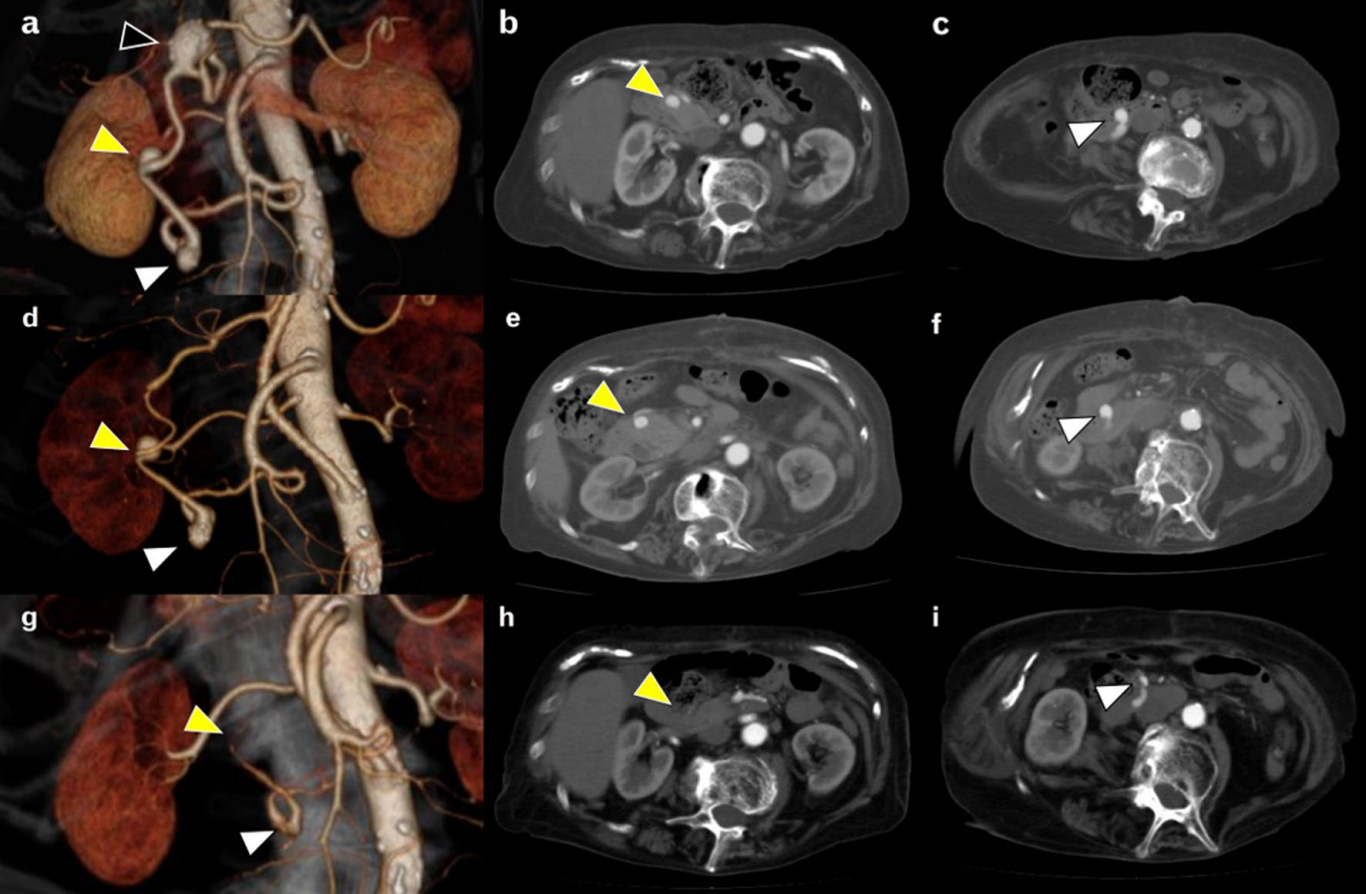
Three-dimensional computed tomography and contrast-enhanced computed tomography. **a** Three-dimensional computed tomography (3D-CT) before bypass surgery revealed a 20 × 17 mm superior pancreaticoduodenal artery aneurysm (SPDAA) (black arrowhead), 10 × 10 mm inferior pancreaticoduodenal artery aneurysm (IPDAA) (yellow arrowhead), 11 × 11 mm IPDAA (white arrowhead), and dilated pancreaticoduodenal arcade (PDA). **b** 10 × 10 mm inferior pancreaticoduodenal artery aneurysm (yellow arrowhead). **c** 11 × 11 mm inferior pancreaticoduodenal artery aneurysm (white arrowhead). **d–f** 3D-CT and CT 1 month after bypass surgery and aneurysmectomy revealed regression of the IPDAAs and a decrease in the dilated PDA arcade. **g–i** One year after surgery revealed complete regression of IPDAAs with progression of thrombosis and a decrease in blood flow. Blood flow and aneurysms were mostly diminished

A 68-year-old woman with epigastric mass was referred to our hospital. The patient’s medical history included rheumatoid arthritis. During the routine follow-up for rheumatoid arthritis, abdominal ultrasonography incidentally identified an epigastric hypoechoic mass by her family physician. Contrast-enhanced computed tomography (CT) revealed three PDAAs with occlusion of the celiac axis and a dilated PDA arcade: 20 × 17 mm SPDAA, 11 × 11 mm IPDAA, and 10 × 10 mm IPDAA (Figs. 1,2a–c).

Surgical and endovascular treatment of IPDAAs is difficult because of the anatomical difficulty and shape of aneurysms. Although SPDAA was resectable, IPDAA was difficult to perform aneurysmectomy. There was a risk of high invasion when considering pancreaticoduodenectomy to resect IPDAAs. Endovascular embolism also carries the risk of total occlusion of the hepatic artery blood flow caused by celiac axis occlusion because of the shape of the IPDAAs. Therefore, we planned a two-stage hybrid therapy. First, we performed an aorto-splenic bypass with a 5-mm prosthetic graft and resection of the SPDAA to preserve hepatic artery blood flow. We could not identify the celiac root, which was almost completely defective. There was no median arcuate ligament during surgery. We encircled the splenic artery and aorta proximal to the inferior mesenteric artery. We then performed a side-to-end anastomosis of the prosthetic graft (WL Gore & Associates, Flagstaff, Arizona, USA) to the splenic artery by running sutures with Gortex CV-6 suture (WL Gore & Associates, Flagstaff, Arizona, USA). The middle of the pancreas and mesentery was mobilized to throw the prosthetic graft. We clamped the aorta partially and performed side-to-end anastomosis of the prosthetic graft, throwing the mobilized space to the aorta by running sutures with CV6. We performed an aneurysmectomy of the gastroduodenal artery aneurysm and end-to-end anastomosis using interrupted 6-0 polypropylene suture (Ethicon, Somerville, New Jersey, USA). On postoperative day 10, the patient was discharged from our hospital without any complications. We then scheduled coil embolization of the IPDAAs; however, the follow-up CT before endovascular embolization showed that the IPDAAs decreased and the dilated PDA arcade improved a month after surgery (Fig. 2d–f). Therefore, we decided to continue a careful follow-up. One year after the operation, the size of the IPDAAs had completely regressed, and the blood flow in the PDA arcade had significantly decreased (Fig. 2g–i). She is doing well without graft occlusion, and the PDAAs have completely regressed 7 years after surgery.

## Discussion

PDAA accounts for only 2% of visceral artery aneurysms but has a high mortality rate [1]. Previous studies have reported that the mortality rate of ruptured PDAA is 21–26% [10, 11]. Therefore, PDAA should be treated adequately in the early stages; however, diagnosis is often difficult because it is asymptomatic before rupture and is diagnosed incidentally [2, 7]. Therapeutic strategies are selected based on the shape and location of the PDAA [6, 12]. In this study, we present a case of triple PDAAs with celiac axis occlusion, and aneurysmectomy of SPDAA and aorto-splenic bypass regressed the IPDAAs spontaneously by reducing the high blood flow of the PDA arcade. Therefore, we identified two important clinical issues. Decreasing the hyperinflow to the PDA arcade can lead to regression of the PDAA, and additional endovascular therapy may not be required in all cases when the dilation of the PDA is improved in selected cases.

PDAA can regress spontaneously by reducing the high blood flow in the PDA arcade. Our patient underwent bypass surgery and the PDAAs regressed completely without planned endovascular embolization. A few case reports have reported regression of PDAA, similar to ours [13, 14]. In 2017, Mont et al*.* reported complete regression of PDAA only by undergoing revascularization without operation for ruptured PDAA with median arcuate ligament (MAL) [13]. In 2020, Yamana et al*. *reported regression of PDAA after bypass surgery [14]. They performed endovascular embolization for multiple ruptured PDAAs, but the PDAAs increased in size in the early stages [14]. Subsequently, aorta-common hepatic artery bypass and MAL resection were performed, and the size of the PDAA regressed [14]. These reports support that regression of PDAA with celiac axis occlusion can be achieved by the improvement of unusual dynamic hyperinflow into the PDA arcade, as in our case.

Reducing the dynamic hyperblood flow into the PDA arcade is crucial for treating PDAA with celiac axis stenosis or occlusion, and improving the unusual dilation of the PDA arcade after treatment may be a successful sign. In a previous study, volumetric CT analysis revealed that the PDA arcade in PDAA cases was larger than that in gastroduodenal artery aneurysm cases [15]. PDA arcade dilation is an important characteristic of its pathogenesis [15]. In our case, PDAAs were caused by celiac arterial axis occlusion. There was no evidence of risk factors such as Marfan syndrome or Behçet’s disease, although other vasculitis and genetic investigations were not investigated in this case. The pathological findings revealed partial intimal thickening and atherosclerotic changes in the media without evidence of segmental arterial mediolysis. There were some options for the bypass for our case, such as aorto-splenic artery, iliac artery–hepatic artery, aorto-hepatic artery, and SMA–hepatic artery. For our case, we chose the aorto-splenic artery bypass using a retroperitoneal approach. This approach helps to maintain stable blood flow and prevent graft kinking. We also considered the possibility of pancreaticoduodenectomy if coil embolization for multiple PDAAs fails. Thus, we performed an aorto-splenic bypass to reduce abnormal inflow into the PDA arcade and support celiac artery blood flow into the hepatic artery. Previous reports suggested that reducing the PDA arcade blood flow may be sufficient to treat PDAA with celiac arterial axis stenosis [16]. Other reports have revealed that PDA arcades undergo an initial expansion phase, followed by the formation of focal aneurysms that have the potential to rupture. To the best of our knowledge, we have reviewed CT and angiography images in case reports published within a few decades. In most cases that can be assessed both before and after treatment, the dilated PDA arcade seemed to be normalized after successful treatment for celiac root stenosis or occlusion by revascularization or bypass [6, 8, 13, 14, 17, 18].

We must carefully follow-up and understand the risk of rupture because PDDA has the potential to rupture even if the size is small. The association between aneurysm size and the risk of rupture has not been clarified. Bageacu et al. reviewed the true PDAAs cases, and the sizes of the ruptured and unruptured aneurysms were 4–30 mm and 5–18 mm, respectively [17]. Takao et al*.* reported that true PDAAs might have a lower rupture risk than expected [19]. They followed six unruptured and untreated PDAA aneurysms with celiac axis stenosis or occlusion, and there was no rupture or size increase, but only one anterior pancreaticoduodenal artery aneurysm increased in size without rupture during 45 months of follow-up [19]. Therefore, novel criteria or assessments are required to predict whether additional endovascular interventions should be performed for PDAA and to assess rupture risk.

Evaluating the risk of PDAA rupture can be difficult using current methods; however, recent research has provided helpful information to accurately assess the risk of aneurysms. High aneurysm wall enhancement (AWE) values are associated with late sac shrinkage after endovascular repair of abdominal aortic aneurysms [20]. This study revealed a significant association between the AWE value on postoperative days 4–7 and the rate of sac shrinkage (*R*^2^ = 0.0139) [20]. In addition, other studies have quantified hemodynamic alterations in the PDA arcade in cases of celiac artery stenosis using computational simulations [21]. Numerical predictions indicate that the arterial network structure can be altered by the blood flow [21]. Furthermore, they also suggested with an electronic circuit model that severe celiac artery stenosis could cause drastic changes in blood flow increase in the PDAA [22]. It can predict the redistribution of blood inflow into the PDA arcade and hepatic artery and may be applied to evaluate the risk of PDAA rupture after treatment. These data support the establishment of criteria to predict the prognosis of PDAA before and after treatment.

## Conclusions

Here, we describe a case of SPDAA and two IPDAAs with celiac axis occlusion that spontaneously regressed after aneurysmectomy for SPDAA and aorto-splenic bypass. Normalization of the hyperinflow to the PDA arcade can lead to regression of the PDAA. Additional endovascular therapy may not be required when PDA dilation improves. However, more cases must be accumulated to establish criteria for predicting the risks of short- and long-term PDAA ruptures.

## Data Availability

The dataset supporting this article is available upon reasonable request from the corresponding authors.

## Abbreviations

AWE: Aneurysm wall enhancement
CT: Computed tomography
MAL: Median arcuate ligament
PDA: Pancreaticoduodenal artery
PDAA: Pancreaticoduodenal artery aneurysm
SPDAA: Superior pancreaticoduodenal artery aneurysm
IPDAA: Inferior pancreaticoduodenal artery aneurysm
